# Morphological Structure and Distribution of Hairiness on Different Body Parts of *Apis mellifera* with an Implication on Pollination Biology and a Novel Method to Measure the Hair Length

**DOI:** 10.3390/insects13020189

**Published:** 2022-02-11

**Authors:** Kamal Ahmed Khan, Tengteng Liu

**Affiliations:** College of Life Sciences, Shandong Normal University, Jinan 250014, China; 2019200017@stu.sdnu.edu.cn

**Keywords:** hairiness, pollination efficiency, morphology of hairiness, western honey bee, evolution

## Abstract

**Simple Summary:**

The western honey bee (*Apis mellifera*) is an important ecological pollinator that commercially and economically contributes billions of dollars of free services to our ecosystem. Having the basic knowledge to provide an effective and standardized measure of the trait and check the pollination effectiveness is very important. However, we are still lacking efficient methods that can significantly measure traits. In this study, we checked the morphological structure of hairs on all of the body parts of the western honey bee by using a scanning electron microscope (SEM); furthermore, we also described the slide preparation method to measure hair length, which could also be used for distinct invertebrates. We found two forms of long and short hairs along with five different types of branches. These branches are worthwhile in pollination effectiveness during holding and transporting pollen. The thoracic region is filled with long hairs with maximum branches, which suggests that the thorax in honey bees probably plays a significant role in pollination effectiveness.

**Abstract:**

Bees play a very important role in pollination, especially western honey bees, which contribute upwards of billions of dollars concerning crop pollination. Hairiness plays an important role in pollination success by transporting pollen, and pollen intake, but there is a lack of detailed studies on the morphological mechanisms. The hairiness trait is barely discussed in pollinator trait analysis because of the lack of systematic techniques used to measure hairiness. This paper reports a novel method that is used to measure the hair length of different body parts of a western honey bee through a stereomicroscope equipped with live measurement module software. Scanning electron microscope (SEM) was used to update the knowledge regarding the hair structure of a western honey bee. We explained different types of hairs, hair branches, and their distributions on different body parts, which are discussed in detail. A positive correlation was found between hair length and the number of branches on all body parts. Five types of branches were observed, and these branches vary with different body parts. Our study provides sufficient details about the hair morphology of the western honey bee and a new methodology for measuring hair length. This methodology will improve the knowledge about understanding the pollination efficiency of the western honey bee.

## 1. Introduction

Pilosity is an important trait in insect pollinators [1]. Hairy forelegs in honey bees are essential for pollen removal and are also used to remove pollen from the eyes, and spacing in hairs is also beneficial in this process [2]. These hairs on bees are beneficial and perfectly adopted to collect pollen [3]. Hairiness plays an important role in thermoregulation by alleviating the convective loss of heat [4,5,6].

A functional trait can be morphological, phonologic, biological, or behavioral features of an organism that influence its health or performance [7]. Morphological traits are significantly important in certain habitats and under certain conditions when compared with other traits. If the species with specific traits and also responses towards changes in the environment are missed, this can cause alteration in an ecosystem following an intense reaction to human-dependent services [8]. The measurement of some traits containing animal functional diversity, mostly in terrestrial invertebrate communities, is deficient [9], or is measured by using methods that are not effective and also have limitations [1].

Bees act as key regulators and also maintain global biodiversity and food security. Honey bees provide pollination services that are important for a variety of crops. Western honey bees (*Apis mellifera* Linnaeus, 1758) and eastern honey bees (*Apis cerana* Fabricius, 1793) have higher pollination efficiency as compared to wasp species (Vespidae) and hoverfly species (Syrphidae) [10]. Honey bees also play an important role in nourishing food security and biodiversity around the world [11].

Pollinators are declining [12,13], including bees across Europe [14,15] and bumblebees in North America [16], facing extreme threats due to climate change, land conversion, excessive use of pesticides, invasive alien species, pollinator’s management, and pathogens [14,17]. These factors have caused a reduction in pollination services provided by plants [15,18].

The western honey bee pollinates wild plants and crops [19,20], acting as a significant key pollinator. The foraging range of honey bees is 2.5–3 km and has not been affected by the landscape context up to 750 m [21]. During the past 5000 years, western honey bees have been misused by humans [22,23] because the colonies of western honey bees and Asian honey bees offer commercial pollination that is exploited and commercialized as managed pollinators [22]. Honey bees are under pressure due to pathogens and environmental stress [20]. Hairiness plays an essential role in collecting pollen and increasing the pollination efficiency, and therefore studies should be conducted focusing on pollinators’ hairiness. We selected the western honey bee as a model bee, however the same study can be conducted on other insect species.

Most studies about western honey bees consider this species as an essential pollinator [24,25], helpful in transporting pollens [26]. The colonies of honey bees can be easily moved from one place to another to collect honey, however pollination is now becoming more profitable than income from honey [27]. The western honey bees are considered an effective pollinator in crop pollination, contributing an estimated value of $ 5–14 billion in the USA per year [27,28].

Pollinators play a very important role in our ecosystem as 87% of the important global food crops are dependent upon animal pollination [29]. In Maoxian County, Sichuan, China, people use sticks and brushes to pollinate apple plants, which reveals a scarcity of pollinators to pollinate fruit plants [30].

Past studies intended to measure hairiness in bees and other insects that rely on the live measurement of hair length [1] and morphology of hairs were described on the thoracic part [4]. These approaches are effective, but usually hair jumbling can cause an error during measurement and hair morphology was not illustrated on whole body parts. Our approach uses the SPM (slide preparation method), which is considered to be a more precise method to measure hair length. An innovative method was proposed to measure entropy attained from the image of the insect body surface as a proxy of hairiness [31], but this was denied by Roquer-Beni et al. [1], declaring that shiny cuticles yielded a high level of entropy because of light reflection. We used a dead specimen of a western honey bee for our measurement, but live (anesthetized) insects can also be used without affected by light reflection. Here, we explain this method to examine the hair structure on different body parts of the honey bee. This study aimed to check the hair structure on different body parts of the honey bee and develop a new method, the slide preparation method (SPM), to measure the hair length. We observed a lack of basic information about hair structure; therefore, this study will contribute towards a better understanding of the importance of hairiness in insect ecology.

## 2. Materials and Methods

**Figure 1:**
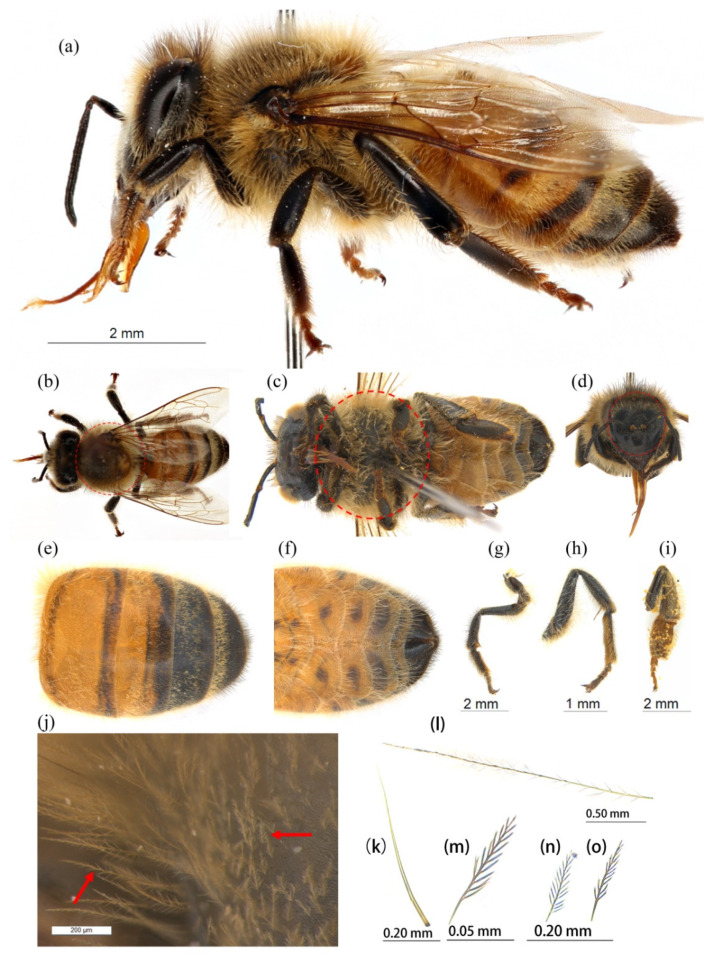
Body parts are used to remove hairs for analysis. (**a**) Pinned western honey bee, (**b**) dorsal thorax, (**c**) ventral thorax, the surface of the ventral thorax is shown in red circle. (**d**) face, (**e**) dorsal abdomen, (**f**) ventral abdomen, (**g**) foreleg, (**h**) mid-leg, (**i**) hind leg, and (**j**) dorsal thorax, red arrows indicate hair branches. (**k**–**o**) Photos taken from the slide by using the LEICA M165C stereomicroscope (Leica Microsystems Ltd., Heerbrugg, Switzerland), (**k**) hairs without any branch, and (**l**–**o**) with branches.

We selected the adult western honey bee (*Apis mellifera*), collected from the city park of Jinan, PR China (Figure 1), because of its importance as a key pollinator. A stereomicroscope, LEICA M165C (Leica Microsystems Ltd, Heerbrugg, Switzerland), was used to take photographs of different body parts and the Hitachi TM3030 environmental scanning electron microscope (SEM, Hitachi High-Technologies Corporation, Hitachi, Japan) was used to capture ultra-photos of hairs. Photoshop^®^ CS6 (Adobe Systems, San Jose, California, CA, USA) was used to improve the quality of the photos.

### 2.1. Hair Morphology

**Figure 2:**
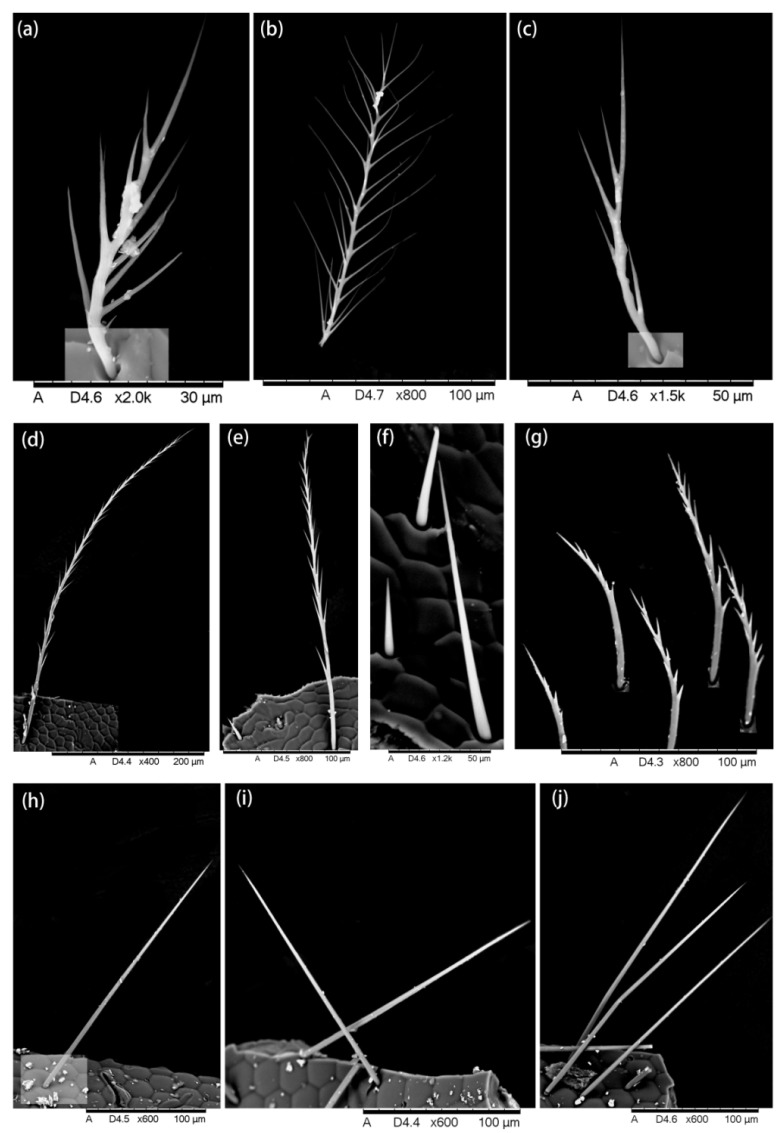
(**a**–**g**) Hairiness structure on the face, (**b**) the long branches and (**d**) short branches, (**h**–**j**) compound eye, hairs without branches are shown.

**Figure 3:**
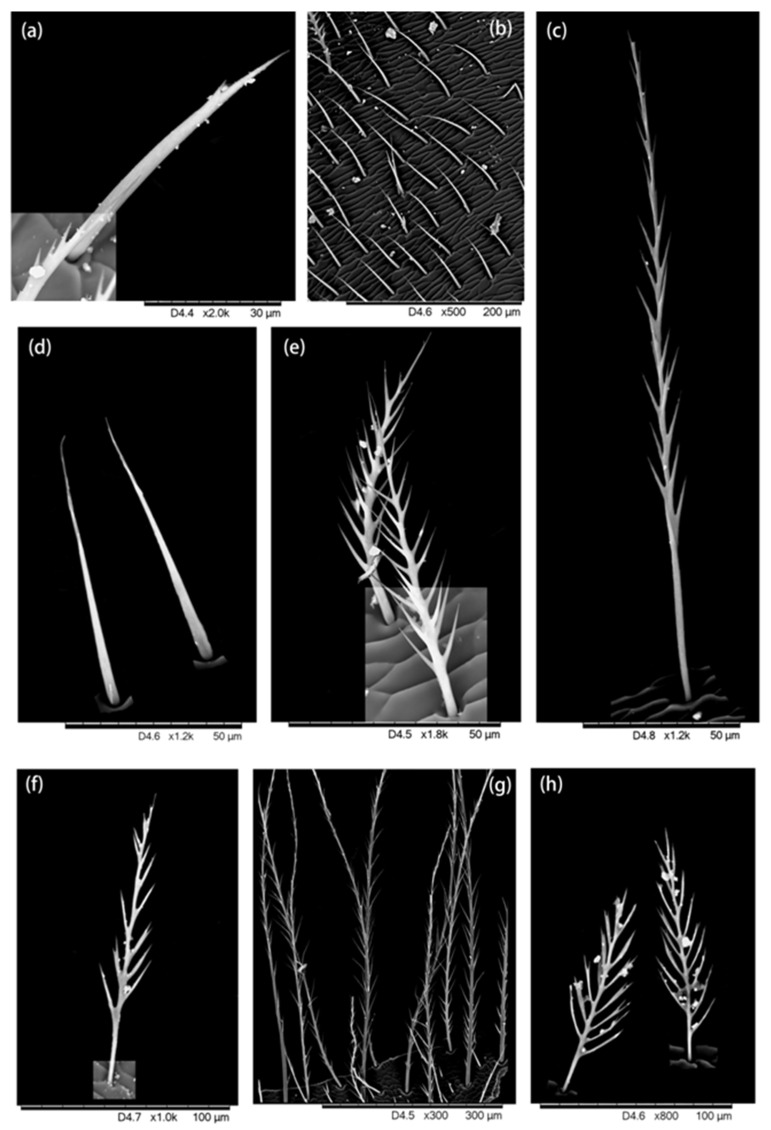
Hairiness structure on the thorax. (**a**–**e**) dorsal thorax: (**a**) hairs with very short branches, (**b**,**d**) short hair without branches, (**c**) long hairs with short branches, (**e**) short hairs with long branches. (**f**–**h**) Ventral thorax, two distinct forms of hairs were found on the ventral thorax: (**f**,**h**) short hairs with multiple branches, and (**g**) long hairs with short branches.

**Figure 4:**
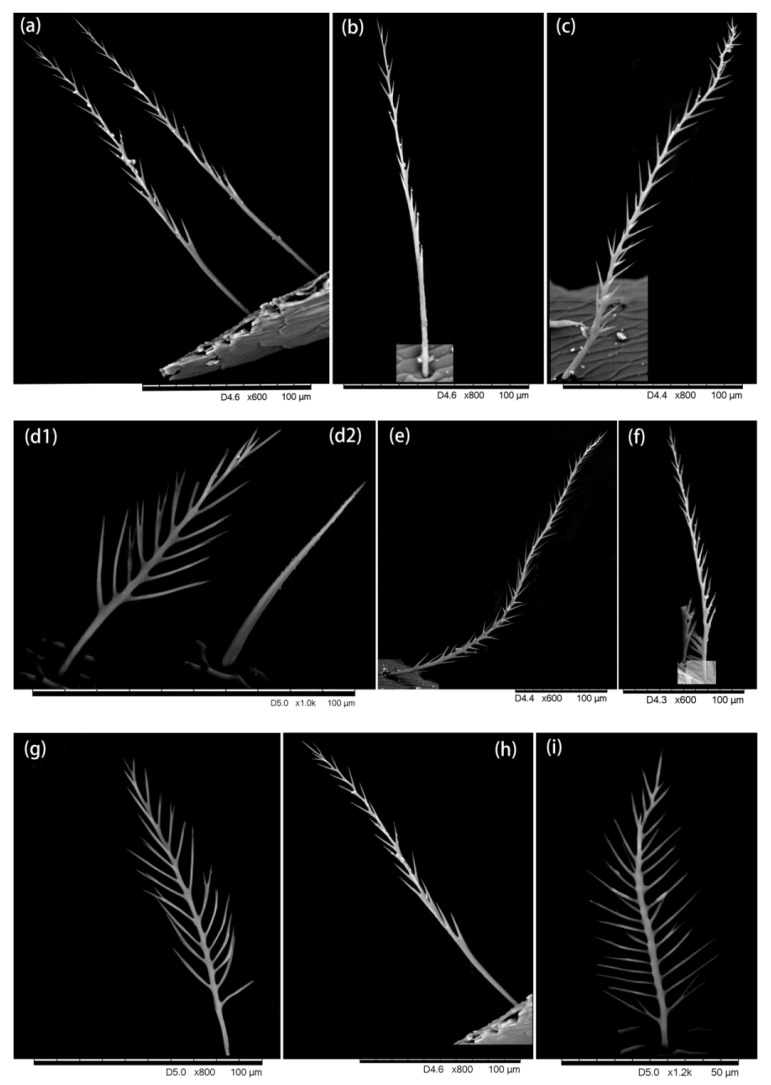
Hairiness structure on the abdomen. (**a**–**e**) Dorsal abdomen: (**a**–**c**,**e**) long hairs with short branches, (**d1**) short hair with multiple branches and (**d2**) short hair without branches. (**f**–**i**) Ventral abdomen, (**f**,**h**) long hair and short branches, and (**g**,**i**) short hairs with multiple branches.

**Figure 5:**
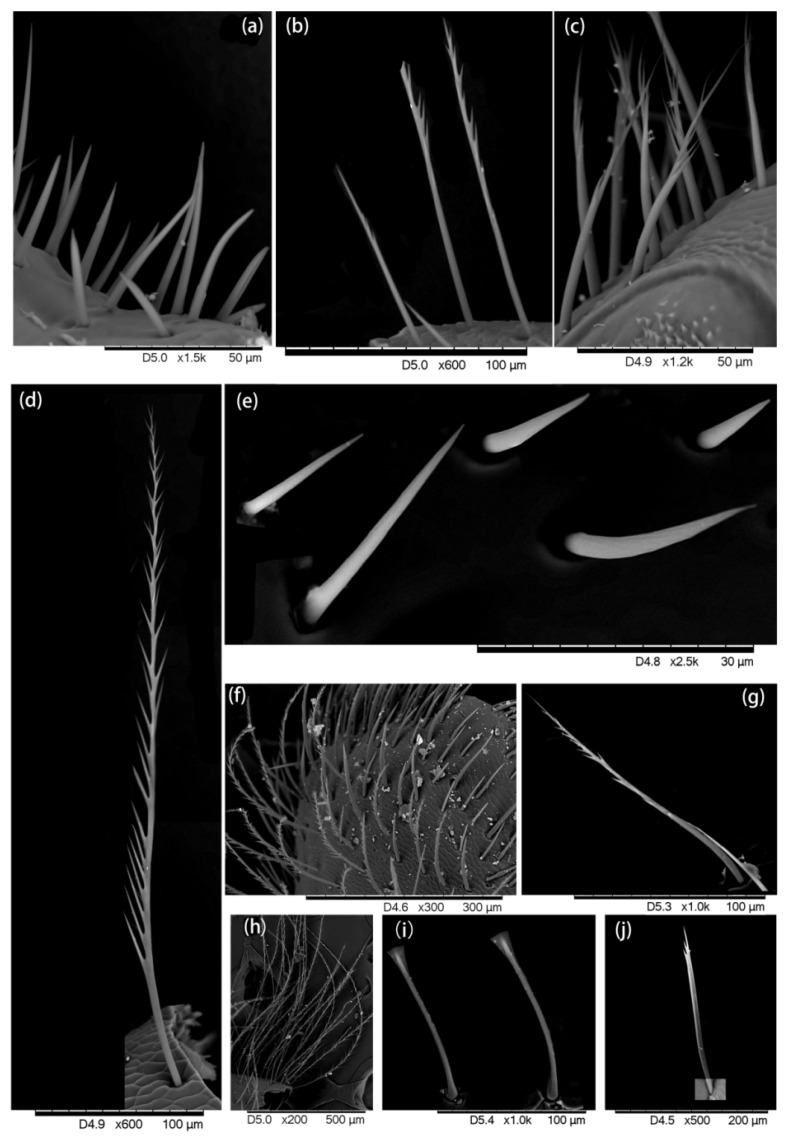
Hairiness structure on the leg. (**a**–**e**) Coxa, short and thin hairs without branches, (**b**,**c**) very few branches at the end of the hair, (**d**) long hair with short branches. (**f**–**h**) Trochanter, (**f**,**g**) three forms of hair, including (**f**,**h**) long hairs with short branches, (**g**) short hairs with short branches, and (**f**) short hairs without branches, were found. (**i**,**j**) Femur, two different forms are found: one end with a triangular structure (**i**) and other ends with very short branches (**j**).

**Figure 6:**
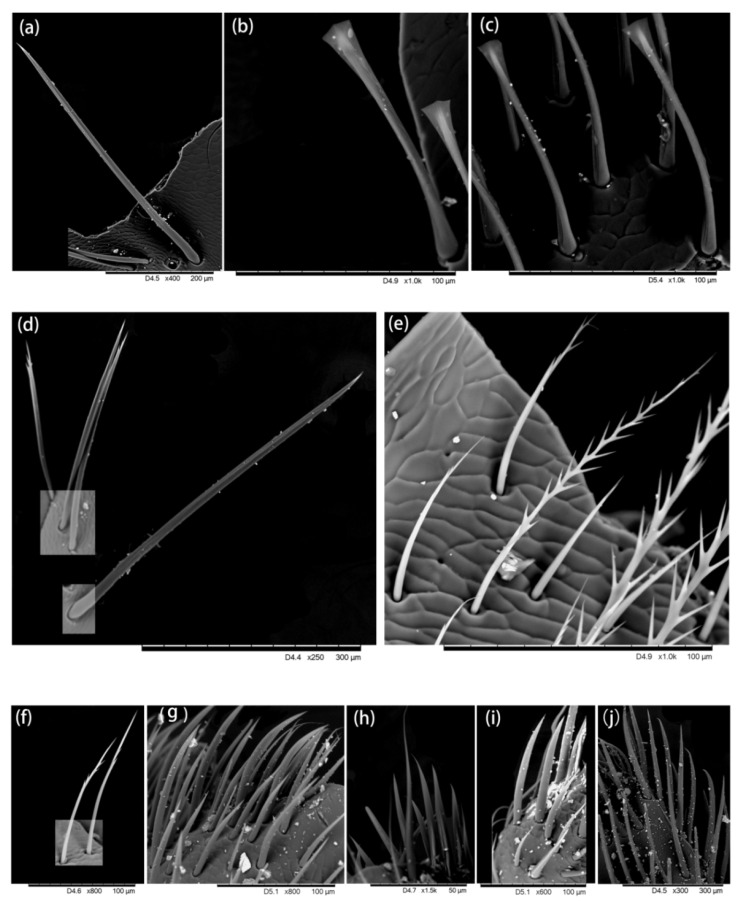
Hairiness structure on the hind leg. (**a**–**d**) Femur: (**a**,**d**) short hairs without branches (**b**,**c**), short hairs without branches and end with triangular structure. (**e**,**f**) Tibia, short hairs with small branches and hairs without branches. (**g**–**j**) Basitarsus, short hairs without branches.

A graver (an instrument with sharp removable blades) was used to cut small tissues from desired parts and prepare the slides for the Hitachi TM3030 environmental SEM. The hairs attached to each tissue were observed and ultra-micro-photos were taken which showed the hair morphology (Figure 2, Figure 3, Figure 4, Figure 5 and Figure 6).

For the use of SEM, tissues with hairs were removed from different body parts and coated with gold-ion in a Giko IB-5 Ion Coater, with 15 kV voltages [4,32].

Hairiness structure was observed on the face, dorsal thorax, ventral thorax, dorsal abdomen, ventral abdomen, and hind leg. The leg was further observed looking at each segment: the coxa, trochanter, femur, tibia, basitarsus, and tarsus. We also analyzed the thorax and face because the thorax is supportive in producing endogenous heat [5] and the face and thorax are used to exchange pollen during pollination [33].

### 2.2. Slide Preparation Method

The slide preparation method was designed to obtain a more accurate measurement of hairiness: 1 × 3 inches long and 1–1.2 mm thick glass slides were used. The hairs were removed from the desired part of the pinned specimen using the graver and these removed hairs were separated from each other by using needles, and the LEICA S9E stereomicroscope (Leica Microsystems Ltd., Heerbrugg, Switzerland) was used for clear observation of the hairs. A drop of pulverized Arabic gum was added and slowly placed on the coverslip to avoid bubbles against the slide. Desiccate slides were used for taking photos.

### 2.3. Hair Length

Hairs were removed and used to prepare slides: 200 hairs were selected from 20 body parts, the thorax (dorsal, ventral), abdomen (dorsal, ventral), face, foreleg, mid-leg, and hind leg, including five segments (coxa, trochanter, femur, tibia, tarsus) from each leg. The LEICA DM 1000 microscope (Leica Microsystems Ltd., Heerbrugg, Switzerland) was used for observation and taking photos. These photos were taken to measure the hair length while the number of branches was counted live on each hair using a 20× zoom lens. The length measuring tool LAS 4.0 software (Leica Microsystems Ltd., Heerbrugg, Switzerland) in the LEICA M165C stereomicroscope was used to measure the hair length.

### 2.4. Statistical Analysis

All the statistical analyses and graphs were made by using R v.4.1.1 (https://cran.r-project.org/, accessed on 9 August 2021) [34]. Hair length and the number of branches are discussed in the results as Mean ± SE. The Kolmogorov–Smirnov test [35] and Shapiro–Wilk test [36] were used to check the data normality. Square root transformations were applied to improve the normality of data if needed. The Pearson correlation coefficient was used to check the correlation value (r) between hair length and the number of branches among different body parts. We also checked the correlation of hair length and number of branches among different body parts by using one-way ANOVA followed by a Tukey’s test, for multiple comparisons.

## 3. Results

### 3.1. Hair Morphology

**Figure 7:**
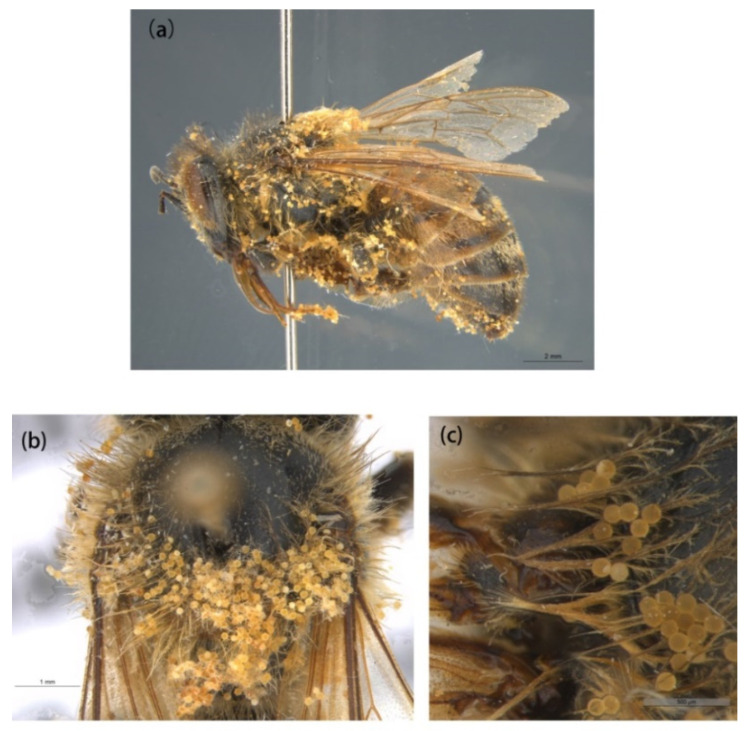
Pollen of Alcea rosea (Malvaceae), attached to body parts of the western honey bee. (**a**) Whole body, and (**b**,**c**) close-up on the dorsal thorax.

We found five types of branches: hairs with long branches (Figure 2a–c), without branches (Figure 2f,h–j), short branches (Figure 2d,e), triangular shape (Figure 5i and Figure 6b,c), and multiple branches (Figure 4g,i), respectively. Two distinct types of short and long hairs followed by five different branches were seen on the body parts. The collected foraging western honey bees were examined using the LEICA M165C stereomicroscope, which showed that pollen attached to hair mostly on the thoracic parts, and it was also found that branches play an important role in holding the pollen (Figure 7).

### 3.2. Correlation between Hair Length and Number of Branches

Hair length and the number of branches were statistically analyzed. The mean and standard error were calculated as shown in Table 1.

The longest hairs and the maximum number of branches were found on the ventral thorax (29.67 ± 1.80 mm) and the basal part of the legs attached to the ventral thorax, especially the coxa. The mean hair length from all body parts was 22.27 mm, while the mean number of branches was 5.23. The minimum hair length was 8.91 mm, while the maximum hair length was 39.60 mm, as shown in Supplementary Table S1.

The average hair length was (16.65 ± 0.92), (24.05 ± 1.25), and (29.67 ± 1.80) on the face, dorsal thorax, and ventral thorax, respectively. The average number of branches was calculated as (4.63 ± 0.15), (4.38 ± 0.33), and (5.85 ± 0.29) on the face, dorsal thorax, and ventral thorax, respectively.

**Figure 8:**
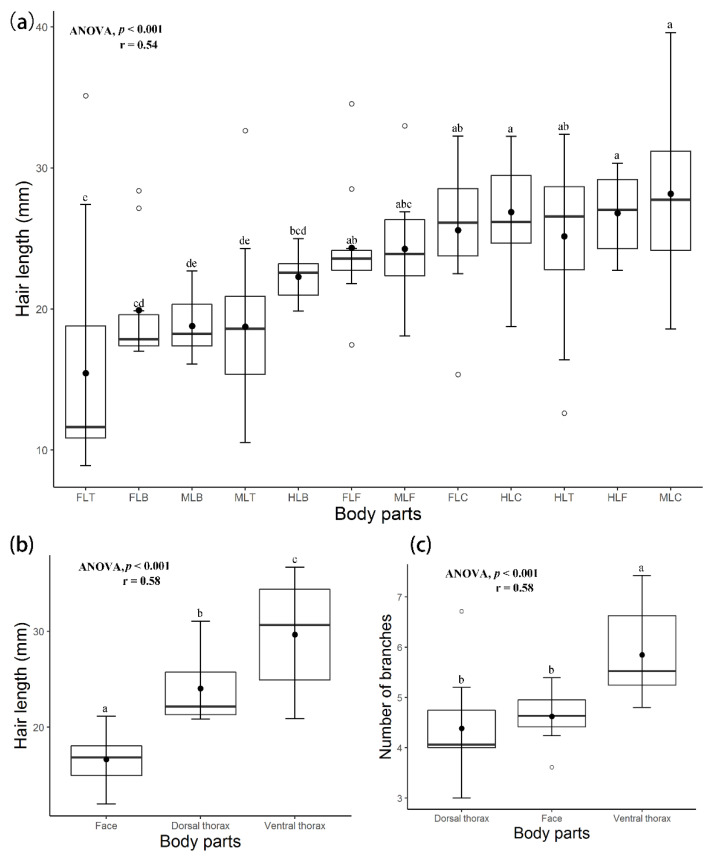
(**a**) The results of hair length and the number of branches on legs, where: FLT, foreleg thorax; FLB, foreleg basitarsus; MLB, mid-leg basitarsus; MLT, mid-leg tarsus; HLB, hind leg basitarsus; FLF, foreleg femur; MLF; mid-leg femur; FLC, foreleg coxa; HLC, hind leg coxa; HLT, hind leg trochanter; HLF, hind leg femur; MLC, mid-leg coxa. (**b**,**c**) Thorax and face. *p*-value shows the significant difference. Treatments with the same letter are not significantly different (one-way ANOVA and Pearson correlation coefficient (r)). Tukey’s test showed that the length across all parts was significantly different, while branches show that treatments with the same letter are not significantly different (*p* > 0.05).

A significant difference in hair length on whole body parts was found (ANOVA, F19 = 10.32, *p* < 0.001) as well as in number of branches (ANOVA, F14 = 4.20, *p* < 0.001). Hair length on the thorax and face showed significant values (ANOVA, F2 = 22.63, *p* < 0.001), as did the number of branches (ANOVA, F2 = 8.6, *p* < 0.001). Hair length on legs was also significant (ANOVA, F14 = 8.99, *p* < 0.001), as well as the number of branches (ANOVA, F9 = 3.14, *p* < 0.001) (Figure 8, Supplementary Table S2).

**Figure 9:**
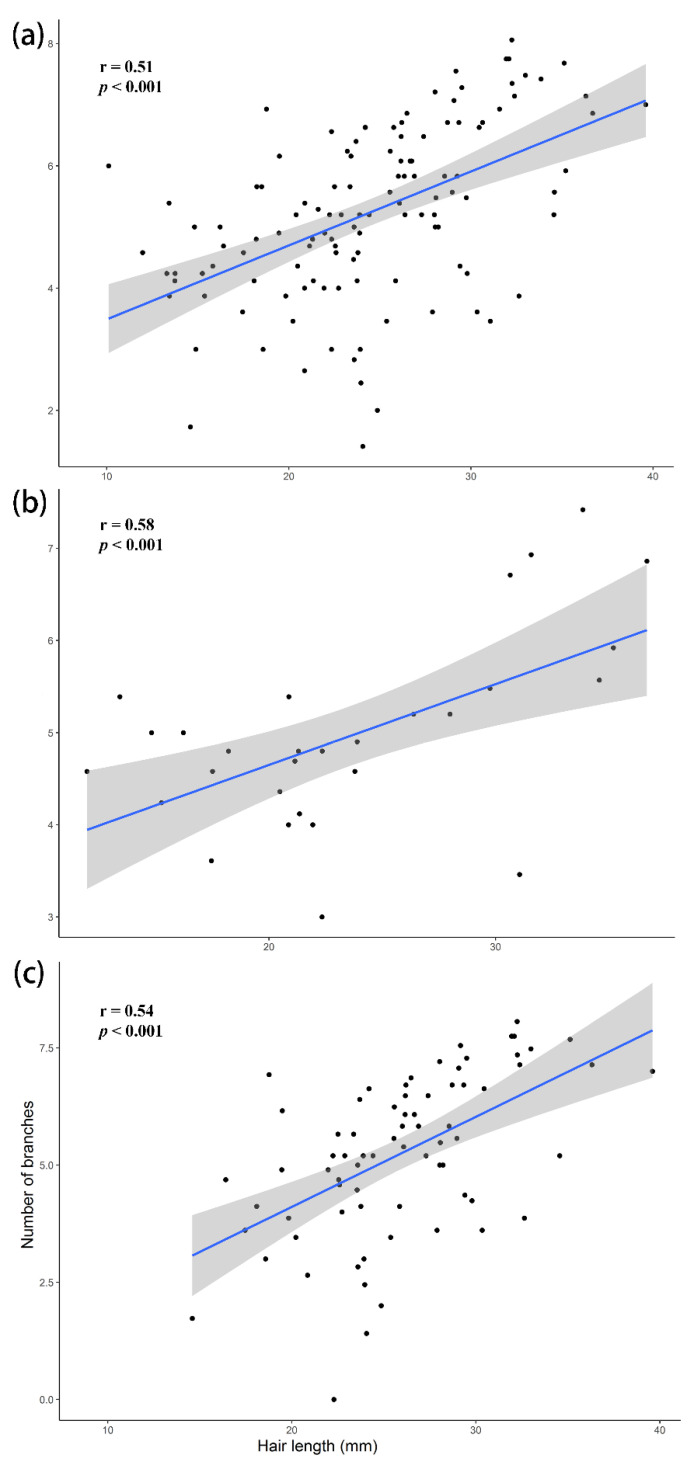
Scatter plots showing the correlation (95% confidence interval of the regression line) between hair length and the number of branches, (**a**) on the whole body parts, (**b**) the face and thorax, and (**c**) the legs.

Scatter plots were used to demonstrate the intercorrelation between hair length and the number of branches. The number of branches and hair length were positively correlated on all body parts (Pearson correlation r = 0.51, *p* < 0.001; Figure 9a). The hair length on all body parts ranged from 8.91 mm (Fore leg trochanter) to 39.60 mm (Ventral thorax) (29.67 ± 1.80), while the number of branches ranged from 0 to 8 (Hind leg coxa) (6.86 ± 0.23). Hair length and the number of branches on the thoracic region and face were positively correlated (r = 0.58, *p* ≤ 0.001; Figure 9b). Hair length and the number of branches on legs, including foreleg, mid-leg, and hind leg, were positively correlated (r = 0.55, *p* < 0.001; Supplementary Table S3, Figure 9c).

## 4. Discussion

To our knowledge, this is the first study that has attempted to explain the morphology of hairs on western honey bees on each body part using a scanning electron microscope (SEM) and measuring hair length by using the slide preparation method (SPM). The goal of our study was to check the morphological structure of hairs and to create a standard method to measure hair length and the number of branches. A distinctive improved method has been described in this study to measure the hair length, and quantitative data of hair length and the associated number of branches were also analyzed. This methodology can also be used for all other pollinators, including butterflies, bees, flies, and beetles, to check the morphology and hair length. The hair length is not affected by the cluttering of hairs, which is a negative factor that was recently described [1].

We found a positive correlation between hair length and the number of branches for all measured body parts (r = 0.51–0.58, *p* < 0.001). Hair length might be affected by body size, as found by Peat et al. [37] in the positive relation between hair length and body size at the intraspecific level in bumblebees.

The results of this study showed the morphological mechanism underlying the association between hairiness and pollination effectiveness. The longer hairs with more branches can hold more pollen grains, and it is significant when related to the size of the pollen grain [38,39], which illustrates significant variations between plant taxa [33]. The outcomes from this study suggest that the western honey bee consists of two forms of hairs that are further divided into five types based on different branches. Interestingly, Phillips et al. [26] and Stavert et al. [31] also found a linkage between hairiness and pollination effectiveness.

The collected specimens were analyzed and it was found that the pollen attached to all body parts, but mostly on the dorsal thorax, face, and legs, especially on the hind leg due to the pollen basket, as shown in Figure 1i and Figure 7. We found a specimen which was full of pollen of *Alcea rosea* (Figure 7) attached to its hairs, and as Mcgregor [40] stated, honey bees visit only one plant species in a single foraging trip and this hairiness enables the bee to accumulate pollen grains on its body that later come in contact with the floral stigma during subsequent bee visits.

We found that the thorax and hind leg have long hairs, and therefore they could play important roles in pollination effectiveness as they can hold more pollen. It was suggested by Southwick [4] that nearly all the pollen was trapped on the periphery by the long hairs. Meanwhile, more branches were found on longer hairs, and this is probably the morphological mechanism for efficiently holding pollen.

Our methodology will be significant for future pollinator studies, looking at their body structure along the environmental gradient and their response to climate change. Researchers suggest that hair length contributes to maintaining the insulation layer and affects thermoregulation [6], and therefore pollinators would have long hairs in cold environments [41]. It is important to remember that the specimens analyzed in the present study were collected from different urban parks of the same city. A larger-scale sampling is necessary in the future to study the genotypic or geographical variation on the morphological traits.

## 5. Conclusions

This study will be useful for measuring hairiness traits such as hair length and morphological structure of hairs on different body parts. We observed the hair structure and measured the hair length using the LAS 4.0 software in LEICA M165C. We found two types of hairs, short hair and long hair, with five different branches. While measuring the hair length, we observed the hair branches and found that different types of hairs have different types of branches. Further research will be useful to find the variations in hair structure and hair length to check the micro-evolution and morphological changes within different insects. 

## Figures and Tables

**Table 1:** Hair length and the number of branches on different body parts of the western honey bee.

Body Parts		Hair Length (mm)	Number of Branches
		Mean ± Standard Error	Mean ± Standard Error
Face		16.65 ± 0.92	4.63 ± 0.15
Thorax	Dorsal surface	24.05 ± 1.25	4.38 ± 0.33
	Ventral surface	29.67 ± 1.80	5.85 ± 0.29
Abdomen	Dorsal surface	15.69 ± 1.25	4.64 ± 0.33
	Ventral surface	23.49 ± 1.30	5.87 ± 0.21
Foreleg	Coxa	25.59 ± 1.48	4.74 ± 0.91
	Trochanter	18.76 ± 2.85	3.03 ± 1.04
	Femur	24.34 ± 1.42	2.70 ± 0.78
	Tibia	12.16 ± 0.84	0
	Basitarsus	19.91 ± 1.34	0
Mid-leg	Coxa	28.17 ± 2.028	4.73 ± 0.80
	Trochanter	21.06 ± 1.57	0.85 ± 0.57
	Femur	24.27 ± 1.31	4.42 ± 0.65
	Tibia	16.43 ± 1.18	1.27 ± 0.58
	Basitarsus	18.90 ± 0.68	0
Hind leg	Coxa	26.88 ± 1.29	6.86 ± 0.23
	Trochanter	26.06 ± 1.54	5.69 ± 0.56
	Femur	26.81 ± 0.89	4.61 ± 0.25
	Tibia	24.25 ± 1.88	0
	Basitarsus	22.28 ± 0.51	0

## Data Availability

Excluded.

## Supplementary Materials

The following supporting information can be downloaded at: https://www.mdpi.com/article/10.3390/insects13020189/s1, Table S1: Summary of data including minimum values, 1st quartile, median, mean, 3rd quartile, and maximum value of hair length and number of branches among the whole body parts. Table S2: Statistical outputs of ANOVA analyzing differences in hair length and the number of branches first with all body parts and then with three body parts (dorsal thorax, ventral thorax, face). Table S3: Correlation coefficients (Pearson r) of hair length and number of branches (dorsal thorax, ventral thorax, face).
