# Supplementary material for: Morphological Structure and Distribution of Hairiness on Different Body Parts of Apis mellifera with an Implication on Pollination Biology and a Novel Method to Measure the Hair Length
Source: Insects. 2022 Feb 11;13(2):189. doi: 10.3390/insects13020189 (PMC8874558; doi:10.3390/insects13020189)
Supplement: Supplementary file 1 [file insects-13-00189-s001.zip › insects-1528623-supplementary.pdf]

Supplementary Materials

Table S1: Summary of data including minimum values, 1st quartile, median, mean, 3rd quartile, and maximum value of hair length and number of branches among the whole body parts.

**Hair length**		**Number of branches**	
Min.	8.91 mm	Min.	0.00
1st Qu.	17.81mm	1st Qu.	4.240
Median	22.43 mm	Median	27.00
Mean	22.27 mm	Mean	5.225
3rd Qu.	26.66 mm	3rd Qu.	6.240
Max.	39.60 mm	Max.	8.060

Table S2: Statistical outputs of ANOVA analyzing differences in hair length and the number of branches first with all body parts and then with three body parts (dorsal thorax, ventral thorax, face).

**Body parts**	**Hair length**			**Number of branches**		
	DF	F	*p* value	DF	F	*p* value
All body parts	19	10.318	2.20×10^{-16} ***	14	4.203	7.53×10^{-6} ***
Face and thorax	2	22.634	1.69×10^{-6}***	2	8.6136	1.28×10^{-3} ***
Legs	14	8.9945	1.19×10^{-13} ***	9	3.1433	3.38×10^{-3}***

***: $p \leq 0.001$

Table S3: Correlation coefficients (Pearson *r*) of hair length and number of branches (dorsal thorax, ventral thorax, face)

**Body part**	**r value**
All	r = 0.51***
Face and thorax	r = 0.58***
Legs	r = 0.54***

***: $p \leq 0.001$
